# Prosthetic Joint Infections Caused by *Mycobacterium tuberculosis* Complex—An ESGIAI–ESGMYC Multicenter, Retrospective Study and Literature Review

**DOI:** 10.3390/microorganisms12050849

**Published:** 2024-04-24

**Authors:** Alvaro Auñon, Llanos Salar-Vidal, Ignacio Mahillo-Fernandez, Francisco Almeida, Pedro Pereira, Jaime Lora-Tamayo, Tristan Ferry, Sarah Souèges, Aurélien Dinh, Rosa Escudero, Candela Menéndez Fernández-Miranda, Alicia Rico, Nicolo Rossi, Jaime Esteban

**Affiliations:** 1 IIS-Fundacion Jimenez Diaz, UAM, Av. Reyes Catolicos 2, 28040 Madrid, Spain; alvaro.aunon@quironsalud.es (A.A.); llanos.salar@quironsalud.es (L.S.-V.); imahillo@fjd.es (I.M.-F.); 2 CIBERINFEC, 28029 Madrid, Spain; sirsilverdelea@yahoo.com (J.L.-T.); rosa.escudero0@gmail.com (R.E.); alirri71@hotmail.com (A.R.); 3 ESCMID Study Group for Implant-Associated Infections (ESGIAI), Aeschenvorstadt 55, 4051 Basel, Switzerland; tristan.ferry@chu-lyon.fr (T.F.); aurelien.dinh@aphp.fr (A.D.); nicorossi89@hotmail.it (N.R.); 4 Hospital de São João, 4200-319 Porto, Portugal; franciscomlrmalmeida@gmail.com (F.A.); pedromferreirapereira@gmail.com (P.P.); 5 Hospital 12 de Octubre, 28041 Madrid, Spain; 6 CHU-Hopital Croix Rousse, 69317 Lyon, France; sarah.soueges@chu-lyon.fr; 7 Ambroise-Paré Hospital, 92104 Paris, France; 8 Hospital Ramon y Cajal, 28034 Madrid, Spain; 9 HUCA, 33011 Oviedo, Spain; candela1987@hotmail.com; 10 Hospital Universitario La Paz, 28046 Madrid, Spain; 11 UOC di Malattie Infettive, Ospedale Guglielmo da Saliceto, AUSL Piacenza, 29121 Piacenza, Italy; 12 Infectious Disease Unit, IRCCS Azienda Ospedaliero Universitaria di Bologna, 40138 Bologna, Italy

**Keywords:** tuberculosis, prosthetic joint infection, treatment, surgery, duration, outcome

## Abstract

Purpose: While tuberculosis remains a significant global health concern, prosthetic joint infections (PJIs) caused by members of the *Mycobacterium tuberculosis* complex are exceptionally rare. Our objective is to perform a retrospective search of new cases of this disease and analyze all cases available in the literature of tuberculous PJIs, aiming to detect factors that may influence patient outcomes. Methods: The ESGIAI and ESGMYC study groups were used to collect information on non-published cases of tuberculous prosthetic joint infections (PJIs). Additionally, a literature review of all published cases of tuberculous PJIs was conducted. All identified cases in the retrospective study and in the literature review were merged and included in the statistical analysis, involving both univariate and multivariate analyses. Results: Fifteen previously unreported cases of tuberculous prosthetic joint infections (PJIs) from four countries were detailed. Among them, ten patients were female, with a median age of 76 years. The hip was affected in 13 cases. Seven patients experienced co-infection with another microorganism. Treatment approaches varied, with 13 patients undergoing implant removal, one treated with DAIR (debridement, antibiotics, and implant retention), and one case was treated with an unknown treatment method. All patients received antibiotic therapy and achieved a cure. The literature review that was conducted detected 155 published cases. Univariate analysis revealed a statistical significance for previous tuberculosis, joint, and no importance of surgery for cure. Conclusions: Tuberculous prosthetic joint infection (PJI) is a rare condition, typically presenting as a localized chronic infection. Antibiotic treatment is essential for the management of these patients, but neither surgical treatment nor duration of treatment seems to have importance in the outcome.

## 1. Introduction

Prosthetic joint infection (PJI) stands as a devastating complication of arthroplasty. This ailment poses a substantial challenge, impacting 1–2% of all arthroplasties. As the number of implanted prostheses escalates, the total number of affected patients correspondingly increases [1,2]. The repercussions of these infections are considerable, resulting in significant costs both at the individual level for patients and at the economic level for the healthcare system.

Tuberculosis continues to be a devastating disease and ranks among the leading causes of death worldwide [3]. Osteoarticular tuberculosis stands as the third most common manifestation of extrapulmonary tuberculosis, constituting approximately 10% of cases [4]. However, *Mycobacterium tuberculosis* is an infrequent cause of prosthetic joint infection (PJI), accounting for about 0.2% of all cases [5]. Despite the main affected structure being the spine, the hip, knee, and shoulder are also affected in high percentages, with all the bones and joints susceptible to infection and disease [4]. Managing these infections remains a significant challenge for various reasons. Firstly, their detection relies on specific tests, emphasizing the importance of considering tuberculosis in the differential diagnosis, as mycobacterial cultures are not typically included in most laboratory protocols. Obtaining a good sample for cultures could be challenging, because they must be obtained through surgical procedures in most cases and, sometimes, samples are not available for diagnosis. Therefore, an indirect approach must be used, including clinical symptoms of the disease, radiological findings, and the positivity of tests for the indirect diagnosis of infection, like PPD or IGRA tests [4]. Given the chronic nature of the clinical disease and its potential minimal signs and symptoms, suspicion of disease is absent until it has fully developed, even with the presence of pathological fractures or vertebral destruction.

Secondly, tuberculous PJI presents in a clinically indistinguishable manner from other chronic infections caused by more common bacterial pathogens. Additionally, in some cases, *M. tuberculosis* may coexist with other faster-growing microorganisms, potentially leading to the underdiagnosis of tuberculosis [6,7,8,9,10]. In these cases, the persistence of symptoms, despite a correct treatment and disease progression, usually lead to the search for unusual pathogens, like mycobacteria. Finally, the diagnosis of these infections often experiences significant delays, due to factors such as the slow growth of the microorganism, resulting in systemic complications [9,11,12,13]. One unanswered question revolves around the necessity of prosthesis removal and the appropriate duration of treatment required for patient cure. Some data suggest that *M. tuberculosis* can form biofilms [14,15], but clear recommendations are currently lacking in existing guidelines, because of the lack of clinical evidence in the literature. Currently, our understanding of the epidemiology, clinical aspects of the disease, and outcomes associated with this condition is limited and is primarily based on case reports or small cohorts with fewer than ten patients. In this study, we present the findings of a multicenter, retrospective investigation conducted by the European Study Group of Implant-Associated Infections (ESGIAI) and the European Study Group of Mycobacteria (ESGMYC). The aim was to encompass a larger number of cases. Additionally, we conducted a literature review of all published cases, aiming to perform a complete statistical analysis that can include all available cases.

## 2. Material and Methods

### 2.1. Retrospective Study

This retrospective, multicenter observational study was conducted collaboratively by the ESGIAI and the ESGMYC. Patients were eligible for inclusion if they were diagnosed with prosthetic joint infections, according to the EBJIS criteria [16], and had a positive culture of *Mycobacterium tuberculosis* complex or a positive molecular detection for *M. tuberculosis* complex from osteoarticular samples between 1990 and 2019. Isolation or PCR detection of *M. tuberculosis* complex in sterile samples (biopsies, implants, and synovial fluid) was considered diagnostic for infection. Inclusion criteria mandated a minimum follow-up period of 2 years for inclusion in the statistical analysis and no control patients were included.

Exclusion Criteria:

(1) Patients with isolation of *M. tuberculosis* complex from samples not related to the affected joint (e.g., respiratory samples) and lacking positive microbiological/pathological results for the prosthetic joint.
(2) Patients with isolation of *M. tuberculosis* complex in samples obtained during the prosthesis implantation (i.e., patients with bone tuberculosis demonstrated by culture during the prosthesis implantation).
(3) Patients with a positive PCR for *M. tuberculosis* complex detected in samples unrelated to the affected joint.
(4) Patients with a positive test for tuberculous infection (Mantoux, IGRA) and PJI, without a positive result from joint samples.
(5) Patients with insufficient follow-up.

The survey for the patients available for the study included demographics (age, sex, and country of origin), underlying conditions (smoke, gastrectomy, liver cirrhosis, COPD, diabetes mellitus, HIV, other immunosuppressive conditions, chronic renal failure, previous tuberculosis, Mantoux test, IGRA, rheumatoid arthritis, and medication used at the time of diagnosis), characteristics of the infected implant (date of implantation, joint, type of prosthesis, tumor prosthesis, and type of fixation), diagnostic tests performed (Serum CRP, Serum ESR, synovial fluid leucocytes, synovial fluid PMN%, histology, thoracic radiography, WBC scintigraphy, bones can, leukocyte esterase, alpha-defensin, and D-dimer), type of surgery (DAIR, one-stage exchange, two-stage exchange, and others), microbiology (isolated species, number of positive samples, antibiogram (first and second line drugs), stain, molecular detection, and other bacterial isolates), antibiotic treatment (antibiotics and length of treatment), and outcome (cure, relapse, reinfection, and failure).

Patients were anonymized and all data were treated according to European and local Data Protection Regulations (RDPG and LOPDGDD 3/2018 in Spain). Ethical approval was obtained from the coordinator center (reference EO128-20_FJD).

### 2.2. Literature Review

A comprehensive literature review was conducted using the PubMed database with the following search criteria: ((*Mycobacterium*) OR (tuberculosis)) AND ((Prosthetic joint infection) OR (prosthetic joint) OR (implant-associated infection)). Additional articles were identified by reviewing the references of articles found in the PubMed search and consulting the relevant textbooks on tuberculosis. We meticulously examined all the references containing data on patients with tuberculous PJI, incorporating the available information into our analysis. No limits on publication time, language, or others were set, with the aim of locating all published cases. We exclude those cases with confirmed diagnosis of tuberculosis at the moment of primary prosthesis implantation. The selected data for analysis included sex, age, prior tuberculosis, psoas abscess, disseminated tuberculosis, joint, time from surgery, treatment, length of treatment, species, surgery (type), and outcome.

### 2.3. Statistical Analysis

The primary endpoint focused on evaluating the efficacy of surgery in achieving patient cure during the follow-up period. Secondary endpoints included assessing the impact of antibiotic treatment on this outcome, identifying the most commonly affected joints, and examining variations in infection characteristics among species within the *Mycobacterium tuberculosis* complex.

A descriptive analysis of the sample was conducted. Qualitative variables were presented as frequencies and percentages, while quantitative variables were summarized using mean and standard deviation or median and quartiles, depending on the data distribution. Two groups were defined based on the outcome (Cure/No cure) and were compared, as concerns the collected variables. Cases of bad outcome, because of other causes not related to tuberculosis, or those that were lost in the follow-up were not included in this analysis. Comparisons between the Cure and No cure groups were made by analyzing the association between each variable and the group. This was carried out using univariable logistic regression models, taking the group as the response (coded 0/1) and taking each of the variables as predictors or explanatory variables. Variables with a *p*-value less than 0.25 in the univariable models were selected as candidates to build the multivariable model. We followed the following steps:

Step 0: the stepwise procedure starts with a model that only includes the intercept term.

Step 1: *p* univariable models are fitted with each of the candidate variables. The statistical significance of each variable is tested and the model with the variable that has the smallest *p*-value is chosen. If this *p*-value is less than 0.05, the process continues; otherwise, it ends.

Step 2: *p*-1 models are fitted, adding each of the *p*-1 remaining variables. The statistical significance for each new variable in the model is tested and the model that includes the variable with the smallest *p*-value is chosen. If the *p*-value for the new variable is less than 0.05, the process continues; otherwise, it ends.

Step 3: new models are fitted, adding each of the variables not included yet. The statistical significance of the variables is tested and the model that includes the variable with the smallest *p*-value is chosen. Now, a backward elimination if performed, deleting those variables with a *p*-value greater than 0.05.

Step 4: is identical to step 3. This process continues until all *p* variables have entered in the model or all variables in the model have a *p*-value less than 0.05; all variables not included in the model have a *p*-value greater than 0.05.

The statistical significance of variables was tested using the likelihood ratio test.

## 3. Results

### 3.1. Retrospective Survey Data

The study included fifteen patients from nine hospitals across four countries (Table 1). Among them, 10 were female, ranging from 47 to 87 years in age, with a median age of 76 years. Four patients had a history of previous tuberculosis. Five individuals were tested for latent tuberculosis, diagnosed through two Mantoux and four IGRA tests (one patient underwent both), yielding positive results in four cases.

In 13 cases, the affected joint was the hip, while in 2 cases, it was the knee. The median time between prosthesis implantation surgery and the development of the disease was 10 months (with a range of 1–132 months). Two patients had revision prostheses, while 13 had primary prostheses. Among the primary prostheses, eight were cemented, five were uncemented, and data were unavailable for two cases. Upon presentation, the median C-reactive protein value was 75 mg/L, ranging from 6.2 to 326.0 mg/L. Thoracic radiography indicated probable tuberculosis in three out of fourteen patients. Treatment approaches included one-stage exchange for eight patients, DAIR (debridement, antibiotics, and implant retention) for two patients, two-stage exchange for two patients, and resection arthroplasty for one patient. One patient received non-surgical treatment and there were no available data regarding the surgical therapy for another patient.

Cultures yielded *Mycobacterium tuberculosis* in 10 patients, while 2 patients had isolates identified as *M. tuberculosis* complex. One patient tested positive for *M. tuberculosis* complex through PCR and, in two cases, positive acid-fast stains were detected, indicating a compatible pathology. Additionally, other microorganisms were detected in cultures taken during the same surgery in six cases, including two cases of *S. aureus*, two cases of *S. epidermidis*, one case of *P. aeruginosa*, one case of *E. coli*, and one case of *Corynebacterium* sp.

All patients received treatment for tuberculosis with a combination of antituberculous drugs, with the most frequently used ones being isoniazid, pyrazinamide, and rifampin (10 patients each), as well as ethambutol (7 patients). One patient had to discontinue isoniazid due to hepatotoxicity. The median treatment duration was 12 months. One patient was lost to follow-up and another experienced re-infection with another microorganism (*Proteus* sp.). One patient passed away one month after surgery, due to reasons unrelated to tuberculosis. This patient was not treated because the culture results became available only after their death. All other patients recovered without complications.

### 3.2. Literature Review

This literature review encompassed 91 references, comprising 155 patients with prosthetic joint tuberculosis. (Supplementary Material contains the available data from these cases and a list of all the reviewed references). Sex was not available in five cases. Of all other cases, 72 were female. Patients have a mean age of 66.8 years. Forty have a previous history of tuberculosis in the past, while 113 did not (two cases do not report this data). Disseminated tuberculosis was reported in 24 cases, but psoas abscess was described only in 1 case. The hip was the most commonly affected joint (96 cases), followed by the knee (52 cases). The mean time from surgery was 67.8 months. Antibiotic treatment was available in 127 cases. All but three of these patients were treated with antibiotics in combinations of two or more, with the most frequent ones being isoniazid in 123 cases and rifampin in 122 cases. The combination of isoniazid + rifampin + ethambutol + pyrazinamide was used as initial treatment in 45 cases; isoniazid + rifampin + ethambutol, with no other antibiotic, was used in 28 cases; isoniazid + rifampin + pyrazinamide was used in 22 cases; isoniazid + rifampin was used in 7 cases; and other combinations were used in 22 patients. Interestingly, no patient was treated with a combination that does not include any of the first-line antituberculous agents (isoniazid, rifampin, ethambutol, or pyrazinamide). In 12 patients, a quinolone was part of the treatment. The length of the therapy was extremely variable, with periods ranging between 5 months and 39 months, but only in 33 cases was the length of therapy below 10 months (including 4 cases that died during the treatment).

From the patients in which the species were identified, *M. tuberculosis* was the most common one (137 cases), followed by *M. bovis* BCG (17 cases) and *M. bovis* (1 case). In 35 patients, no surgical procedure was performed, while, in all other cases, a surgical procedure was performed (DAIR, 1- or 2-stage exchange, or resection arthroplasty). A total of 127 patients were cured, 2 were lost at the follow-up, 11 patients died (7 from causes unrelated to tuberculosis), 5 suffered a relapse, 1 was considered a therapeutic failure, and 1 received suppressive therapy. A total of 11 patients were included in the No cure group (4 died, 5 relapse, 1 suppression, and 1 therapeutic failure).

### 3.3. Data Analysis

The data obtained from the literature review were integrated with the survey data for a comprehensive statistical analysis, using the data that were common for both databases. The results of this analysis are presented as follows: Table 2 and Table 3 showcase the median/mean values of quantitative data and the percentages of qualitative data, respectively.

Table 4 assesses the relationship between each variable and the outcome. Logistic regression models were employed to examine the potential association between each variable and cure, resulting in the calculation of an odds ratios (OR), their confidence intervals, and *p*-values. The OR indicates the risk or probability of non-cure. In this model, statistical significance was achieved for the length of treatment, prior tuberculosis, knee and shoulder joints in comparison to hip, and surgery.

## 4. Discussion

Our study comprises 15 cases of prosthetic joint infection (PJI) caused by the *M. tuberculosis* complex, marking the largest series reported to date. This surpasses the series by Uhel et al. (13 cases) [7] and Meyssonier (9 cases) [17]. This underscores the rarity of this disease, given that most published studies are confined to case reports or small series with fewer than five cases. The scarcity of reported cases may be attributed to the low diagnostic suspicion for tuberculous PJI. In our series, other microorganisms were identified in six cases and the isolation of *M. tuberculosis* was unexpected, likely due to biopsy samples being cultured for mycobacteria as part of the sample culturing protocol. Interestingly, bone and joint tuberculosis appears as one of the most common forms of extrapulmonary tuberculosis, comprising 10% of all cases of these syndromes [4]. However, most cases of this disease occur in the spine, as vertebral tuberculosis, with the characteristic destruction of the vertebra classically known as Pott’s disease. Among peripheral joints, knee and hip rank as being the most frequently involved, being 37% and 23% of this group (13% and 8% of all forms of musculoskeletal tuberculosis) in a report from Los Angeles County from 1990 to 1995, respectively [4].

Moreover, *M. tuberculosis* is now recognized to form biofilms [15], which are a critical pathogenic factor in prosthetic joint infections, with significant implications for patient management [1]. In vitro studies have indicated that tuberculous biofilms demonstrate tolerance against antituberculous agents [15,18], but the clinical implications of this remain unknown. Mycobacterial biofilms are now considered as one of the key pathogenic factors in the disease of other species of mycobacteria, but not in the case of tuberculosis, where other properties (like intracellular survival) seem to be more important. However, since biofilms are considered essential in the pathogenesis of implant-associated infections, particularly in PJIs, this fact must be considered when dealing with cases of tuberculous PJIs and designing their management strategies.

Antimicrobial therapy plays a crucial role in the management of tuberculosis patients, a principle upheld by both our series and the literature review. Therapeutic regimens for tuberculosis have evolved over the years, as new antibiotics have been discovered, but all of them consistently include several antituberculous drugs that are active against the isolated strain, to avoid the development of resistance and ensure an effective therapy for patients. Over the last 50 years, the recommended treatment of tuberculosis has typically included a combination of isoniazid, rifampin, and pyrazinamide, sometimes with the addition of ethambutol, which are considered first-line antibiotics for tuberculosis management, because they have the highest antibacterial activity with a low rate of secondary effects. Other antibiotics may also exhibit good activity and have been used in the management of tuberculosis when resistance to any of the former antibiotics (especially rifampin) appears. The above cited scheme is still recommended for bone tuberculosis with a change only in the treatment duration (from 6 months to 9–12 months) in many guidelines [4]. In our study, all patients were treated with a minimum of 2 antibiotics, with most of them receiving 3 or more drugs (only 12 cases were treated with only 2 drugs). The selected antibiotics typically include isoniazid, rifampin, ethambutol, and pyrazinamide, with only a few cases requiring second-line antibiotics (Supplementary Table S1). Interestingly, some cases added other antibiotics to the conventional treatment (including quinolones in several cases, for example), while, in all cases, a first-line antibiotic is included. Another crucial consideration is the treatment duration, averaging 13.6 months (Table 2), close to the 12 months recommended in some guidelines for osteoarticular tuberculosis. However, while we detected significant differences using 9 months of treatment as a breakpoint, such differences cannot be found for a breakpoint of 13 months, so it seems that increasing the length of the therapy beyond 13 months is not necessary for a good outcome, but a minimum of 9 months is necessary. However, because the number of cases is low, these results must be considered carefully and, probably, an individualized follow-up of each patient is essential. 

Surgical treatment is traditionally considered essential in bacterial and fungal prosthetic joint infections, aiming to reduce bacterial load and eliminate biofilms adhered to the implant. Most of our patients, along with those reported in the literature, underwent surgical management with various approaches [7]. However, 21.2% of all cases were treated without surgery (Table 3, see Supplementary Material for detailed data) and the statistical difference suggest that a surgical procedure improves the outcome. However, again, the low number of cases (especially those with failure) made it necessary to conduct a multidisciplinary, individualized approach that considers all the variables, in order to perform a surgery and select the type of surgical procedure. In fact, despite the ability of *M. tuberculosis* to form biofilms in vitro, it seems that this factor may not be a fundamental aspect of tuberculosis management cases. The intricate relationship between bacteria and the cellular immune system seems to play a crucial role in the development of infections and, in certain instances, the manifestation of diseases [19]. Recent studies have demonstrated lesion heterogeneity, with host factors playing a crucial role in pathogenesis [20]. Furthermore, no studies on adherence or biofilm development onto the materials used in prostheses have been conducted; the potential for *M. tuberculosis* biofilm development on these surfaces may differ from what is described, as demonstrated in other microorganisms [21]. These factors could provide an explanation for the favorable outcomes observed in some cases, where surgery was not performed.

In these merged cases (including all the literature and our series), 42 of them had a previous history of tuberculosis (Supplementary Material). In these instances, the likely pathogenesis involves hematogenous dissemination followed by bone and/or prosthesis infection. The presentation, in these cases, both from our series and the literature, was notably late, with a median of 2 years from surgery, further supporting the hypothesis of hematogenous dissemination; however, there are cases that appear within the first months following prosthesis implantation and we hypothesize that these could be cases of the prosthesis being implanted in a bone with unnoticed tuberculous disease. Bone lesions, like other extrapulmonary forms of the disease, are considered the result of hematogenous dissemination [22] and not direct inoculation of mycobacteria onto the implant during surgery, as is the case with other prosthetic joint infections. Interestingly, disseminated tuberculosis was found in 14.8% of the cases. Additionally, the hip was the most frequently affected joint, leading, again, to the hypothesis that undiagnosed hip tuberculosis could be the cause of a pathological fracture, necessitating the use of a joint prosthesis. In fact, because knee prostheses are more frequently used than hip ones [23], and hip prostheses are more frequently infected in our study, it seems that a special predilection of tuberculosis for this joint can be defined. Subsequently, these prostheses became infected by *M. tuberculosis*, and a chronic infection developed after several weeks. This hypothesis, coupled with the use of prostheses, also accounts for the advanced age of most patients and the extended period between surgery and disease development. In fact, other potential sources of tuberculous infection of the hip, such as a psoas abscess, were detected in only one case.

The etiology is also of interest. Most cases have been caused by *M. tuberculosis*, the most common species of the *M. tuberculosis* complex responsible for human infections, which was the only species isolated in our patients (refer to Table 3). Interestingly, the second leading cause of these infections is *M. bovis* BCG, accounting for approximately 10% of all reported cases. This strain is used as a vaccine worldwide and is also employed in the treatment of vesical cancer, as an immunomodulator, through intravesical inoculation [24]. One of the potential side effects is the hematogenous dissemination of the mycobacterium, with some cases resulting in disseminated disease. This dissemination can lead to a miliary form of the disease, or to a localized form if the mycobacteria reach a susceptible tissue. Our cases are likely secondary to this dissemination, with the strain reaching the joint and causing a prosthetic joint infection through a hematogenous route. The low prevalence of disease caused by wild-type *M. bovis* in developed countries explains the low percentage of PJIs caused by this species, being surpassed in prevalence by the vaccine strain, because of the previously explained reasons.

There are limitations to our study, with the main challenge arising from the extended period over which cases have been published, making it difficult to locate some data in the reports. This may have led to potential inaccuracies in data collection, due to absent or unreliable records in older patient reports. The retrospective nature of the study also contributed to some missing data in the clinical charts and the voluntary participation in the survey can lead to a bias (particularly as the majority of participating countries are from the Mediterranean area). Another important limitation is the relatively low number of reviewed cases and the scarcity of reports in the literature, which limits the robustness of our statistical study. In addition, the low number of patients with a “No cure” outcome limits the statistical analysis, due to the significant disparity between the numbers of patients with good or bad outcomes related to the tuberculous disease. We believe that, for future reviews, it is necessary to publish data on patients with adverse outcomes, because this is a very uncommon disease, making it challenging to conduct clinical studies. Nevertheless, this review presents the most extensive series of cases ever described and reviews the literature, providing valuable insights based on nearly all available cases of tuberculous PJIs.

In conclusion, tuberculous prosthetic joint infection (PJI) is an extremely rare condition, predominantly observed in elderly individuals with hip prostheses (and less frequently, with knee prosthesis), resulting in chronic PJIs. The optimal treatment for these cases remains elusive, but the role of surgery in their management does not appear to be indispensable for a cure. Instead, the use of the recommended combinations of first-line antibiotics is the most commonly employed approach and is probably effective against most cases of the disease.

## Figures and Tables

**Table 1:** Description of the patients included in this study.

Sex	Age	Prior TBC	Joint	Time from Surgery (Months)	Treatment * (Months)	Species	Surgery	Outcome
F	54	No	Hip	48	REPL (6)	*M. tuberculosis*	DAIR	Cure
M	74	Yes	Knee	60	IREP (2) + IR (6)	*M. tuberculosis*	2-stage revision	Cure
M	76	Yes	Hip	96	IREL (3) + IR (12)	*M. tuberculosis*	DAIR	Cure
M	47	No	Hip	1	N/A	*M. tuberculosis*	1-stage revision	Cure
M	87	No	Hip	10	IREP	*M. tuberculosis*	No	Dead **
F	76	No	Hip	2	IRP (2) + IR (7)	*M. tuberculosis*	Resection arthroplasty	Cure
F	86	Yes	Hip	2	IRP (2) + IR (9)	*M. tuberculosis*	No	Cure
F	64	Yes	Knee	3	IREP (2) + IR (6)	*M. tuberculosis*	1-stage revision	Cure
F	87	No	Hip	3	IREPAL	*M. tuberculosis*	DAIR	Cure
F	70	No	Hip	6	IREP (2) + 1R (10)	*M. tuberculosis*	DAIR	Cure
F	81	No	Hip	12	N/A	*M. tuberculosis*	1-stage revision	Cure
F	59	No	Hip	N/A	N/A	N/A	1-stage revision	Cure
F	71	No	Hip	N/A	N/A	N/A	1-stage revision	Cure
M	81	No	Hip	132	IREP (3) + RP (5)	*M. tuberculosis*	2-stage revision	Cure
F	82	No	Hip	60	IRPL (4) + IP (8)	*M. tuberculosis*	2-stage revision	Cure

F: Female, M: Male. *: I: Isoniazid; R: Rifampin; E: Ethambutol; P: Pyrazinamide; L: Levofloxacin; A: Amikacin. DAIR: debridement, antibiotics, and implant retention. N/A: Not available. **: Patient died due to unrelated causes.

**Table 2:** Quantitative variables of all the cases.

Variable	N	Mean (SD) or Median (Quartiles)	Range
Age	164	67.9 (14.5)	(22, 92)
Months of treatment	131	13.6 (6.4)	(1, 39)
Time from surgery	167	24 (6, 84)	(1, 492)

**Table 3:** Qualitative variables of all cases.

Variable	N (%)
**Sex**	
Female	82 (49.7%)
Male	83 (50.3%)
**Prior TBC**	
No	124 (73.8%)
Yes	44 (26.2%)
**Psoas abscess**	
No	161 (99.4%)
Yes	1 (0.6%)
**Disseminated TBC**	
No	138 (85.2%)
Yes	24 (14.8%)
**Joint**	
Hip	109 (64.1%)
Knee	54 (31.8%)
Shoulder	5 (2.9%)
Elbow	1 (0.6%)
Wrist	1 (0.6%)
**Species**	
*M. tuberculosis*	150 (89.3%)
*M. bovis* BCG	17 (10.1%)
*M. bovis*	1 (0.6%)
**Surgery**	
No	37 (21.8%)
Yes	133 (78.2%)
**Type of surgery**	
1-Stage revision arthroplasty	5 (2.9%)
2-Stage revision arthroplasty	30 (17.6%)
Arthrodesis	1 (0.6%)
DAIR	32 (18.8%)
Girdlestone	3 (1.8%)
No surgery	37 (21.8%)
Resection arthroplasty	30 (17.6%)
Revision arthroplasty	31 (18.2%)
Staged exchange	1 (0.6%)
**Outcome**	
Cure	141 (87.0%)
No cure	21 (13.0%)

**Table 4:** Statistical analysis of all cases (univariant studies).

Variable	Cure (n = 141)	No Cure (n = 11)	OR (95% CI)	*p*-Value
**Age**	67.4 ± 14.8	70.1 ± 9.49	1.01 (0.97, 1.07)	0.566
**Months of treatment**	14.1 ± 6.25	11.4 ± 7.45	0.92 (0.79, 1.04)	0.215
**Time from surgery**	24 (7, 84)	31 (7.5, 36)	1.00 (0.98, 1.00)	0.478
**Sex**				
Female	71 (51.8%)	4 (40.0%)	Reference	
Male	66 (48.2%)	6 (60.0%)	1.61 (0.44, 6.55)	0.474
**Prior TBC**				
No	104 (74.8%)	5 (45.5%)	Reference	
Yes	35 (25.2%)	6 (54.5%)	3.57 (1.01, 13.1)	0.046 *
**Psoas abscess**				
No	136 (99.3%)	10 (100%)	Reference	
Yes	1 (0.7%)	0 (0.0%)	NA	
**Disseminated TBC**				
No	119 (86.9%)	7 (70.0%)	Reference	
Yes	18 (13.1%)	3 (30.0%)	2.83 (0.57, 11.3)	0.156
**Joint**				
Hip	94 (66.7%)	2 (18.2%)	Reference	
Knee	42 (29.8%)	9 (81.8%)	10.1 (2.47, 68.0)	0.004 *
Shoulder	3 (2.1%)	0 (0.0%)	NA	
Elbow	1 (0.7%)	0 (0.0%)	NA	
Wrist	1 (0.7%)	0 (0.0%)	NA	
**Species**				
TBC	125 (89.9%)	10 (90.9%)	Reference	
BCG	14 (10.1%)	1 (9.1%)	0.89 (0.05, 5.21)	0.917
BOV	0 (0.0%)	0 (0.0%)	NA	
**Surgery**				
No	26 (18.4%)	5 (45.5%)	Reference	
Yes	115 (81.6%)	6 (54.5%)	0.27 (0.08, 1.00)	0.043 *
Months of treatment				
≥13	45 (39.8%)	8 (50.0%)	Reference	
>13	68 (60.2%)	8 (50.0%)	0.66 (0.23, 1.92)	0.441
Months of treatment				
≥9	97 (85.8%)	10 (62.5%)	Reference	
<9	16 (14.2%)	6 (37.5%)	3.64 (1.11, 11.3)	0.027 *

*: Statistically significant. Reference: values used as reference for the analysis.

## Data Availability

All the data are available in the Supplementary Table S1 and in the Table 1.

## Supplementary Materials

The following supporting information can be downloaded at: https://www.mdpi.com/article/10.3390/microorganisms12050849/s1, Table S1: Data of patents obtained from the cases published in the literature.
