# Supplementary material for: Prosthetic Joint Infections Caused by Mycobacterium tuberculosis Complex—An ESGIAI–ESGMYC Multicenter, Retrospective Study and Literature Review
Source: Microorganisms. 2024 Apr 24;12(5):849. doi: 10.3390/microorganisms12050849 (PMC11123809; doi:10.3390/microorganisms12050849)
Supplement: Supplementary file 1 [file microorganisms-12-00849-s001.zip › microorganisms-2892681-supplementary.pdf]

| Sex | Age | Prior TBC | Psoas Abscess | Miliary TBC | Joint | Time From Surgery | Treatment | Months of treatment | Species | Surgery | Outcome | Reference |
|---|---|---|---|---|---|---|---|---|---|---|---|---|
| M | 80 | No | No | No | Hip | 132 | IRE (15) | 15 | BCG | DAIR | SUPRESSION | [1] |
| F | 73 | No | No | No | Knee | 8 | IREP (9)+IRP (3) | 12 | TBC | DAIR | CURE | [2] |
| F | 75 | No | No | No | Shoulder | 3 | IRP (2)+IR (7) | 9 | TBC | REVISION ARTHROPLASTY | CURE | [3] |
| M | 37 | No | No | No | Elbow | 14 | IREP (6) | 6 | TBC | 2-STAGE REVISION ARTHROPLASTY | CURE | [4] |
| M | 61 | No | No | No | Hip | 24 | | | TBC | RESECTION ARTHROPLASTY | CURE | [5] |
| M | 80 | No | No | Yes | Knee | 96 | IREP (2)+IR (10) | 12 | TBC | NO | CURE | [6] |
| F | 52 | No | No | No | Hip | 120 | IR (15) | 15 | TBC | REVISION ARTHROPLASTY | CURE | [7] |
| F | 44 | No | No | No | Hip | 276 | IE (16) | 16 | TBC | RESECTION ARTHROPLASTY | CURE | [7] |
| F | 52 | Yes | No | No | Hip | 24 | IRE (6) + IE (18) | 24 | TBC | DAIR | CURE | [7] |
| M | 74 | Yes | No | No | Hip | 36 | IRE (3) + IR (9) + I (12) | 24 | TBC | DAIR | THERAPEUTIC FAILURE | [7] |
| F | 55 | Yes | No | No | Hip | 24 | IRES(6) + IR (33) | 39 | TBC | 2-STAGE REVISION ARTHROPLASTY | CURE | [7] |
| M | 60 | Yes | No | No | Hip | 12 | IRE(7)+IE(2)+I(3) | 12 | TBC | RESECTION ARTHROPLASTY | CURE | [7] |
| M | 60 | No | No | No | Hip | 360 | IRE (1)+IR (18) | 19 | TBC | RESECTION ARTHROPLASTY | CURE | [7] |
| M | 65 | No | No | No | Knee | 1 | | | TBC | NO | CURE | [8] |
| F | 55 | No | No | No | Hip | 24 | IREP (4)+IR (13) | 17 | TBC | NO | CURE | [9] |
| F | 47 | Yes | No | No | Hip | 36 | IREP (3)+IR (3) | 6 | TBC | REVISION ARTHROPLASTY | CURE | [9] |
| M | 70 | No | No | No | Hip | 12 | IREP (2)+IR (6) | 8 | TBC | 2-STAGE REVISION ARTHROPLASTY | CURE | [10] |
| M | 77 | Yes | No | Yes | Hip | 18 | RES (7) | 7 | TBC | REVISION ARTHROPLASTY | CURE | [11] |
| F | 72 | No | No | No | Knee | 96 | IRP (24) | 24 | TBC | ARTHRODESIS | CURE | [12] |
| M | 79 | No | | | Knee | 120 | | | TBC | REVISION ARTHROPLASTY | | [13] |
| F | 46 | No | No | No | Hip | 4 | IRP (8) | 8 | TBC | NO | NO DATA | [14] |
| M | 74 | No | No | No | Hip | 30 | | | TBC | RESECTION ARTHROPLASTY | CURE | [15] |
| F | 40 | No | No | No | Hip | 8 | | | TBC | RESECTION ARTHROPLASTY | CURE | [15] |
| M | 75 | Yes | No | No | Hip | 4 | IRP (2)+IR (10) | 12 | TBC | REVISION ARTHROPLASTY | CURE | [16] |

| | | | | | | | | | | | | |
|---|---|---|---|---|---|---|---|---|---|---|---|---|
| M | 68 | No | No | No | Wrist | 24 | IREP (2)+IR (12) | 14 | TBC | REVISION ARTHROPLASTY | CURE | [17] |
| F | 92 | No | No | No | Hip | 36 | IRE (1)+IE (8), L | 9 | TBC | REVISION ARTHROPLASTY | CURE | [17] |
| F | 72 | No | No | No | Hip | 84 | IRP (2)+IR (10) | 12 | TBC | 2-STAGE REVISION ARTHROPLASTY | CURE | [17] |
| F | 79 | No | No | No | Hip | 1 | IRE (5) | 5 | TBC | 2-STAGE REVISION ARTHROPLASTY | CURE | [17] |
| F | 80 | No | No | No | Knee | 1 | IRP (2)+IR (12) | 14 | TBC | 2-STAGE REVISION ARTHROPLASTY | DIED (OTHER CAUSES) | [17] |
| M | 77 | No | No | No | Knee | 96 | IREP (2)+IRE (22) | 24 | BCG | 2-STAGE REVISION ARTHROPLASTY | CURE | [18] |
| F | 75 | Yes | No | No | Knee | 3 | IREP (9) | 9 | TBC | DAIR | CURE | [19] |
| F | 67 | Yes | Yes | No | Hip | 16 | IREP (2)+IR (18) | 20 | TBC | DAIR | CURE | [20] |
| F | 69 | No | No | No | Hip | 12 | | | TBC | RESECTION ARTHROPLASTY | CURE | [21] |
| F | 71 | No | No | No | Hip | 12 | IRE (18) | 18 | TBC | NO | CURE | [21] |
| F | 22 | Yes | No | No | Hip | 36 | IRE (19) | 19 | TBC | DAIR | CURE | [21] |
| F | 64 | No | No | No | Hip | 48 | | 18 | TBC | DAIR | CURE | [21] |
| F | 75 | No | No | No | Hip | 120 | | | TBC | RESECTION ARTHROPLASTY | CURE | [21] |
| F | 77 | No | No | Yes | Knee | | IRP | | TBC | NO | CURE | [22] |
| M | 73 | No | No | No | Knee | 14 | IREP (2)+IR (10) | 12 | TBC | REVISION ARTHROPLASTY | CURE | [23] |
| F | 54 | No | No | No | Hip | 48 | REPL (6) | 6 | TBC | DAIR | CURE | Present study |
| M | 74 | Yes | No | No | Knee | 60 | IREP (2)+IR (6) | 8 | TBC | 2-STAGE REVISION ARTHROPLASTY | CURE | Present study |
| M | 76 | Yes | No | No | Hip | 96 | IREL (3)+IR (12) | 15 | TBC | DAIR | CURE | Present study |
| M | 47 | No | No | No | Hip | 1 | | | TBC | 1-STAGE EXCHANGE | CURE | Present study |
| M | 87 | No | No | No | Hip | 10 | IREP | | TBC | NO | DIED (OTHER) | Present study |
| F | 76 | No | No | No | Hip | 2 | IRP (2)+IR (7) | 9 | TBC | RESECTION ARTHROPLASTY | CURE | Present study |
| F | 86 | Yes | No | No | Hip | 2 | IRP (2)+IR (9) | 11 | TBC | NO | CURE | Present study |
| F | 64 | Yes | No | No | Knee | 3 | IREP (2)+IR (6) | 8 | TBC | 1-STAGE EXCHANGE | CURE | Present study |
| F | 87 | No | No | No | Hip | 3 | IREPAL | | TBC | DAIR | CURE | Present study |
| F | 70 | No | No | No | Hip | 6 | IREP (2)+1R (10) | 12 | TBC | DAIR | CURE | Present study |

| | | | | | | | | | | | | |
|---|---|---|---|---|---|---|---|---|---|---|---|---|
| F | 81 | No | No | No | Hip | 12 | | 12 | TBC | 1-STAGE EXCHANGE | CURE | Present study |
| F | 59 | No | No | No | Hip | | | 14 | | 1-STAGE EXCHANGE | CURE | Present study |
| F | 71 | No | No | No | Hip | | | 12 | | 1-STAGE EXCHANGE | CURE | Present study |
| M | 81 | No | No | No | Hip | 132 | IREP (3)+RP (5) | 8 | TBC | 2-STAGE REVISION ARTHROPLASTY | CURE | Present study |
| F | 82 | No | No | No | Hip | 60 | IRPL (4)+IP (8) | 12 | TBC | 2-STAGE REVISION ARTHROPLASTY | CURE | Present study |
| M | 47 | Yes | No | No | Knee | 18 | IRE (12) | 12 | TBC | DAIR | CURE | [24] |
| M | 78 | No | No | No | Hip | 6 | IRE (3)+IR (6) | 9 | TBC | RESECTION ARTHROPLASTY | CURE | [25] |
| M | 67 | No | No | Yes | Knee | 1 | IRE (6)+IR (6) | 12 | TBC | NO | CURE | [26] |
| M | 77 | No | No | No | Hip | 216 | IRE (12) | 12 | BCG | 2-STAGE REVISION ARTHROPLASTY | CURE | [27] |
| M | 82 | No | No | No | Hip | 120 | IREP+IR (12) | 12 | BCG | 2-STAGE REVISION ARTHROPLASTY | CURE | [28] |
| M | 66 | No | No | No | Hip | 60 | IR (6) | 6 | BCG | REVISION ARTHROPLASTY | DIED (OTHER CAUSES) | [29] |
| F | 60 | Yes | No | No | Knee | 7 | IREP (12)+IR (9) | 21 | TBC | REVISION ARTHROPLASTY | CURE | [30] |
| F | 78 | No | No | No | Hip | 5 | IR | | TBC | 2-STAGE REVISION ARTHROPLASTY | CURE | [31] |
| M | 27 | Yes | No | No | Hip | 3 | IE | | TBC | RESECTION ARTHROPLASTY | CURE | [32] |
| M | 71 | Yes | No | No | Hip | 5 | IRE (12), P | 12 | TBC | DAIR | CURE | [33] |
| F | 54 | No | No | No | Hip | 108 | IE | | TBC | RESECTION ARTHROPLASTY | CURE | [34] |
| F | 79 | No | No | No | Knee | 31 | IEP | | TBC | DAIR | RELAPSE | [34] |
| M | 49 | No | No | No | Hip | 120 | IRP+C/O | | TBC | 2-STAGE REVISION ARTHROPLASTY | | [34] |
| M | 83 | No | No | No | Knee | 10 | IRE | | TBC | DAIR | CURE | [34] |
| M | 61 | No | No | No | Hip | 7 | IRE | | TBC | RESECTION ARTHROPLASTY | CURE | [34] |
| F | 48 | Yes | No | No | Hip | 420 | NO ANTIBIOTIC | | TBC | GIRDLESTONE | CURE | [35] |
| F | 51 | Yes | No | No | Hip | 492 | IRE | | TBC | NO | CURE | [35] |
| F | 85 | No | No | No | Knee | 1 | | | TBC | NO | CURE | [36] |
| F | 72 | No | No | Yes | Hip | 108 | IEP | | TBC | RESECTION ARTHROPLASTY | CURE | [37] |

| | | | | | | | | | | | | |
|---|---|---|---|---|---|---|---|---|---|---|---|---|
| F | 75 | No | No | No | Knee | 3 | IE (18), R | 18 | TBC | RESECTION ARTHROPLASTY | CURE | [38] |
| F | 36 | Yes | No | No | Knee | 11 | IREPM (19) | 19 | TBC | 2-STAGE REVISION ARTHROPLASTY | CURE | [39] |
| F | 67 | Yes | No | No | Hip | 2 | IRSP (3)+IR (9) | 12 | TBC | REVISION ARTHROPLASTY | CURE | [40] |
| F | 66 | No | No | No | Hip | 48 | IRP (9), R | 9 | TBC | REVISION ARTHROPLASTY | CURE | [41] |
| F | 73 | No | No | Yes | Shoulder | 2 | IREL (2)+IL (10) | 12 | BOV | RESECTION ARTHROPLASTY | DIED (OTHER CAUSES) | [42] |
| F | 77 | No | No | No | Knee | 36 | IRPM (2)+IR (9) | 11 | TBC | 2-STAGE REVISION ARTHROPLASTY | CURE | [43] |
| M | 84 | No | No | No | Knee | 72 | RE | | BCG | GIRDLESTONE | CURE | [44] |
| M | 74 | No | No | No | Shoulder | 444 | IREP (2)+IR (8) | 10 | TBC | RESECTION ARTHROPLASTY | CURE | [45] |
| F | 79 | No | No | No | Knee | 2 | IREP (12) | 12 | TBC | DAIR | CURE | [46] |
| F | 81 | No | No | Yes | Hip | 48 | IRS (4)+IR (32) | 36 | TBC | RESECTION ARTHROPLASTY | CURE | [47] |
| F | 70 | No | No | No | Hip | 8 | | | TBC | NO | CURE | [48] |
| F | 49 | No | No | No | Hip | 8 | | | TBC | NO | CURE | [48] |
| M | 50 | No | No | No | Hip | 36 | IREP (2)+IR (10) | 12 | TBC | REVISION ARTHROPLASTY | CURE | [49] |
| F | 71 | No | No | No | Hip | 2 | IRE (2)+IR (10) | 12 | TBC | REVISION ARTHROPLASTY | CURE | [49] |
| F | 75 | No | No | No | Knee | 180 | IREP (1)+IEP (4)+IP (1) | 6 | TBC | RESECTION ARTHROPLASTY | DIED | [50] |
| M | 64 | No | No | No | Hip | 3 | IREP (2)+IRE (8)+IR (8) | 18 | TBC | 2-STAGE REVISION ARTHROPLASTY | CURE | [51] |
| F | 71 | Yes | No | No | Hip | 24 | NO ANTIBIOTIC | | TBC | GIRDLESTONE | CURE | [52] |
| M | 66 | No | No | No | Knee | 2 | IRP (6) | 6 | TBC | 2-STAGE REVISION ARTHROPLASTY | CURE | [53] |
| F | 65 | Yes | No | Yes | Knee | 3 | IRP (6) | 6 | TBC | 2-STAGE REVISION ARTHROPLASTY | CURE | [53] |
| F | 77 | No | No | No | Knee | 4 | IEP (8) | 8 | TBC | DAIR | CURE | [53] |
| M | 48 | No | No | Yes | Knee | 9 | IREPM (1) | 1 | TBC | NO | DIED | [54] |
| F | 77 | Yes | No | No | Hip | 84 | IRS (2)+IR (16) | 18 | TBC | DAIR | CURE | [55] |
| M | | No | | | Hip | 1 | | | TBC | NO | | [56] |
| M | 70 | No | No | No | Hip | 144 | IREM (1)+IRE (11) | 12 | BCG | 2-STAGE REVISION ARTHROPLASTY | CURE | [57] |

| | | | | | | | | | | | | |
|---|---|---|---|---|---|---|---|---|---|---|---|---|
| F | 32 | No | No | No | Hip | 60 | ACP (2)+P (2) | 4 | TBC | 2-STAGE REVISION ARTHROPLASTY | CURE | [58] |
| M | 80 | No | No | No | Hip | 12 | IREP (2)+IR (16) | 18 | TBC | NO | CURE | [59] |
| M | 69 | No | No | Yes | Hip | 12 | IREP (2)+IR (16) | 18 | TBC | NO | CURE | [59] |
| F | 64 | No | No | No | Hip | 12 | IREP (2)+IR (16) | 18 | TBC | NO | CURE | [59] |
| F | 83 | No | No | No | Hip | 12 | | | TBC | NO | LOST | [59] |
| M | 56 | Yes | No | No | Hip | 24 | IREP (2)+IR (10) | 12 | TBC | REVISION ARTHROPLASTY | CURE | [59] |
| M | 86 | No | No | Yes | Hip | 42 | IREP (2)+IR (13) | 15 | TBC | NO | CURE | [59] |
| M | 84 | No | No | No | Hip | 84 | IREP (2)+IR (12) | 14 | TBC | 2-STAGE REVISION ARTHROPLASTY | CURE | [59] |
| M | 59 | No | No | Yes | Hip | 156 | IREP (2)+IR (10) | 12 | TBC | REVISION ARTHROPLASTY | CURE | [59] |
| F | 84 | Yes | No | No | Hip | 240 | IREP (2)+IR (13) | 15 | TBC | NO | CURE | [59] |
| M | 37 | No | No | No | Hip | 12 | | | TBC | RESECTION ARTHROPLASTY | | [60] |
| F | 82 | No | No | Yes | Hip | 24 | IREP (2)+IR | | TBC | NO | CURE | [61] |
| F | 73 | No | No | No | Knee | 168 | IREP (4)+IRP (3)+IR (11) | 18 | TBC | NO | CURE | [62] |
| M | 90 | No | No | No | Hip | 372 | IREP (2)+IRE (10) | 12 | BCG | DAIR | CURE | [63] |
| M | 64 | No | | | Hip | 24 | | | TBC | REVISION ARTHROPLASTY | | [64] |
| M | 91 | No | No | No | Hip | 204 | IRE (2)+IR (10) | 12 | BCG | 2-STAGE REVISION ARTHROPLASTY | CURE | [65] |
| M | 84 | No | No | No | Hip | 1 | IREP (2)+IR (7) | 9 | TBC | NO | CURE | [66] |
| F | 82 | No | No | No | Hip | 1 | IRP (2)+IR (2) | 4 | TBC | REVISION ARTHROPLASTY | LOST | [66] |
| M | 86 | No | No | No | Hip | 120 | IRP (6)+IR (6)+ I(12) | 24 | BCG | REVISION ARTHROPLASTY | CURE | [67] |
| M | 66 | No | No | No | Hip | 60 | IR (12) | 12 | BCG | DAIR | CURE | [68] |
| M | 79 | No | No | No | Hip | 18 | IREM (6) | 6 | BCG | 2-STAGE REVISION ARTHROPLASTY | CURE | [69] |
| M | 76 | No | No | No | Knee | 216 | IRE (24) | 24 | BCG | 2-STAGE REVISION ARTHROPLASTY | CURE | [70] |
| | | Yes | | | Hip | 34 | | | TBC | 2-STAGE REVISION ARTHROPLASTY | CURE | [71] |
| | | No | | | Knee | 34 | | | TBC | NO | DIED | [71] |
| | | No | | | Hip | 34 | | | TBC | DAIR | CURE | [71] |

| | | | | | | | | | | | | |
|---|---|---|---|---|---|---|---|---|---|---|---|---|
| | | Yes | | | Hip | 34 | | | TBC | 2-STAGE REVISION ARTHROPLASTY | CURE | [71] |
| | | Yes | | | Hip | 34 | | | TBC | 2-STAGE REVISION ARTHROPLASTY | CURE | [71] |
| F | 59 | No | No | No | Hip | 15 | REP (12) | 12 | TBC | DAIR | CURE | [72] |
| F | 70 | No | No | No | Knee | 54 | RE (12), I | 12 | TBC | DAIR | CURE | [13] |
| M | 76 | No | No | No | Hip | 72 | IRE | 9 | BCG | 2-STAGE REVISION ARTHROPLASTY | CURE | [73] |
| M | 86 | No | No | No | Shoulder | 24 | REL (1) | 1 | BCG | REVISION ARTHROPLASTY | DIED (OTHER CAUSES) | [73] |
| M | 66 | No | No | No | Hip | 324 | IRE (6) | 6 | BCG | REVISION ARTHROPLASTY | CURE | [74] |
| F | 74 | Yes | No | Yes | Knee | 12 | IRE (14) | 14 | TBC | DAIR | RELAPSE | [75] |
| M | 68 | Yes | No | No | Knee | 1 | IRE (14) | 14 | TBC | NO | RELAPSE | [75] |
| M | 70 | Yes | No | No | Knee | 2 | IRE (14) | 14 | TBC | NO | RELAPSE | [75] |
| F | 61 | Yes | No | No | Knee | 6 | IRE (14) | 14 | TBC | NO | RELAPSE | [75] |
| M | 55 | No | No | No | Knee | 1 | IREP (2)+IR (10) | 12 | TBC | NO | CURE | [76] |
| F | 72 | No | No | No | Hip | 36 | IR (12) | 12 | TBC | RESECTION ARTHROPLASTY | | [77] |
| F | 70 | No | No | No | Knee | 456 | IR (12) | 12 | TBC | RESECTION ARTHROPLASTY | CURE | [77] |
| F | 71 | Yes | No | No | Knee | 20 | IE (18) | 18 | TBC | RESECTION ARTHROPLASTY | CURE | [77] |
| M | 62 | No | No | Yes | Hip | 18 | IRP (24) | 24 | TBC | REVISION ARTHROPLASTY | CURE | [78] |
| M | 40 | No | No | Yes | Hip | 180 | IRE (12) | 12 | TBC | RESECTION ARTHROPLASTY | CURE | [78] |
| M | 61 | No | No | No | Hip | 24 | IRE (3)+IR (6) | 9 | TBC | DAIR | CURE | [79] |
| F | 84 | No | No | No | Knee | 36 | IREP (2)+IR (10) | 12 | TBC | NO | CURE | [79] |
| M | 79 | Yes | No | No | Hip | 48 | IREP (1)+REP (3)+RP (11) | 15 | TBC | REVISION ARTHROPLASTY | DIED (OTHER CAUSES) | [79] |
| M | 60 | Yes | No | No | Hip | 60 | IRE (14)+IR (4) | 18 | TBC | NO | CURE | [79] |
| M | 79 | Yes | No | Yes | Knee | 84 | IREP (2)+IR (10) | 12 | TBC | NO | CURE | [79] |
| M | 86 | No | No | No | Knee | 108 | IREO (4)+IRO (2) | 6 | TBC | RESECTION ARTHROPLASTY | CURE | [79] |
| F | 82 | No | No | No | Knee | 108 | IRP (1)+IR (14) | 15 | TBC | RESECTION ARTHROPLASTY | CURE | [79] |
| M | 69 | Yes | No | No | Hip | 120 | IREP (6)+IR (18) | 24 | TBC | REVISION ARTHROPLASTY | CURE | [79] |
| M | 84 | Yes | No | Yes | Knee | 156 | IRE (2)+IR (7) | 9 | TBC | DAIR | DIED (OTHER CAUSES) | [79] |

| | | | | | | | | | | | | |
|---|---|---|---|---|---|---|---|---|---|---|---|---|
| F | 85 | No | No | No | Knee | 168 | IREP (1)+IRE (1)+ IR (10) | 12 | TBC | RESECTION ARTHROPLASTY | CURE | [79] |
| M | 71 | Yes | No | Yes | Hip | 240 | NO ANTIBIOTIC | | TBC | NO | DIED BEFORE DX | [79] |
| F | 81 | No | No | Yes | Hip | 5 | IREP (2)+IR (15) | 17 | TBC | DAIR | CURE | [79] |
| F | 64 | No | No | No | Shoulder | 6 | IRP (2)+IR (30) | 32 | TBC | DAIR | CURE | [79] |
| F | 27 | | NO | NO | Knee | 36 | IREP (2)+IR (16) | 18 | TBC | DAIR | CURE | [80] |
| M | 39 | Yes | No | No | Hip | 48 | MR (31) | 31 | TBC | 2-STAGE REVISION ARTHROPLASTY | CURE | [81] |
| F | 84 | No | No | No | Hip | 6 | IRP (12) | 12 | TBC | REVISION ARTHROPLASTY | CURE | [82] |
| F | 84 | No | No | No | Hip | 4 | IRP (12) | 12 | TBC | REVISION ARTHROPLASTY | CURE | [83] |
| M | 67 | No | No | Yes | Hip | 24 | IRE (18) | 18 | TBC | REVISION ARTHROPLASTY | CURE | [83] |
| M | 61 | No | No | No | Hip | 48 | IRP (18) | 18 | TBC | REVISION ARTHROPLASTY | CURE | [83] |
| M | 35 | No | No | Yes | Hip | 12 | IRE (12) | 12 | TBC | RESECTION ARTHROPLASTY | CURE | [83] |
| M | 62 | No | No | No | Knee | 36 | IRP (2)+IR (16) | 18 | TBC | DAIR | CURE | [84] |
| F | 34 | No | No | No | Knee | 48 | IREP (2)+IR (16) | 18 | TBC | NO | CURE | [84] |
| M | 84 | Yes | No | No | Knee | 5 | IREP (12)+IR (9) | 21 | TBC | REVISION ARTHROPLASTY | CURE | [85] |
| M | 73 | No | No | Yes | Hip | 108 | IREP (9) | 9 | TBC | RESECTION ARTHROPLASTY | CURE | [86] |
| M | 72 | Yes | No | Yes | Knee | 36 | IREP (1) | 1 | TBC | DAIR | DIED | [87] |
| M | 70 | No | No | No | Hip | 108 | IREM (3)+IRM (6)+IR (3) | 12 | BCG | REVISION ARTHROPLASTY | CURE | [88] |
| M | 61 | No | No | No | Knee | 12 | IR (24) | 24 | TBC | STAGED EXCHANGE | CURE | [89] |
| F | 70 | No | No | No | Hip | 4 | | | TBC | NO | | [89] |
| M | 62 | No | No | No | Knee | 1 | IRE (12) | 12 | TBC | NO | CURE | [90] |
| M | 63 | No | No | No | Knee | 1 | IR (12) | 12 | TBC | NO | CURE | [90] |
| F | 40 | | NO | NO | Knee | 48 | | | TBC | RESECTION ARTHROPLASTY | CURE | [91] |

M: Male; F: Female. Prior TBC: Previous diagnosis of tuberculosis. I: Isoniazid; R: Rifampin; E: Ethambutol; P: Pyrazinamide; S: Streptomycin; L: Levofloxacin; M: Moxifloxacin; C: Ciprofloxacin; O: Ofloxacin; A: Amikacin. TBC: *Mycobacterium tuberculosis*; BOV: *Mycobacterium bovis* subsp. *bovis*; BCG: *Mycobacterium bovis* BCG strain. DAIR: Debridement, Antibiotics, and Implant Retention.

References


1. Aitchison LP, Jayanetti V, Lindstrom ST, Sekel R. Myobacterium bovis peri-prosthetic hip infection with successful prosthesis retention following intravesical BCG therapy for bladder carcinoma. Australas Med J 2015;8:307–14. doi: 10.4066/AMJ.2015.2475

2. Al-Shaikh R, Goodman SB. Delayed-onset Mycobacterium tuberculosis infection with staphylococcal superinfection after total knee replacement. Am J Orthop (Belle Mead NJ) 2003;32:302–5.

3. Amouyel T, Gaeremynck P, Gadisseux B, Saab M, Senneville E, Maynou C. Mycobacterium tuberculosis infection of reverse shoulder arthroplasty: a case report. J Shoulder Elbow Surg 2019;28:e271–4. doi: 10.1016/j.jse.2019.04.052

4. Asopa V, Wallace AL. Case report: Management of occult tuberculosis infection by 2-stage arthroplasty of the elbow. J Shoulder Elbow Surg 2004;13:364–5. doi: 10.1016/j.jse.2003.12.012

5. Baldini N, Toni A, Greggi T, Giunti A. Deep sepsis from Mycobacterium tuberculosis after total hip replacement. Case report. Arch Orthop Trauma Surg (1978) 1988;107:186–8. doi: 10.1007/BF00451602

6. Barry M, Akkielah L, Askar MA, Bin Nasser AS. Miliary tuberculosis with delayed-onset total knee arthroplasty Mycobacteria tuberculosis infection successfully treated with medical therapy alone: A case report and literature review. Knee 2019;26:1152–8. doi: 10.1016/j.knee.2019.07.010

7. Berbari EF, Hanssen AD, Duffy MC, Steckelberg JM, Osmon DR. Prosthetic joint infection due to Mycobacterium tuberculosis: a case series and review of the literature. Am J Orthop (Belle Mead NJ) 1998;27:219–27.

8. Besser MI. Total knee replacement in unsuspected tuberculosis of the joint. Br Med J 1980 14;280:1434. doi: 10.1136/bmj.280.6229.1434

9. Bernard L, Arvieux C, Brunschweiler B, Touchais S, Ansart S, Bru J-P, et al. Antibiotic Therapy for 6 or 12 Weeks for Prosthetic Joint Infection. N Engl J Med 2021 27;384:1991–2001. doi: 10.1056/NEJMoa2020198

10. Brown A, Grubbs P, Mongey A-B. Infection of total hip prosthesis by Mycobacterium tuberculosis and Mycobacterium chelonae in a patient with rheumatoid arthritis. Clin Rheumatol 2008;27:543–5. doi: 10.1007/s10067-007-0788-6

11. Bruns J, Luessenhop S, Behrens P. Haematogenous tuberculous infection following revision of a loosened total hip replacement. Langenbecks Arch Surg 1998;383:265–8. doi: 10.1007/s004230050130

12. Bryan WJ, Doherty JH, Sculco TP. Tuberculosis in a rheumatoid patient. A case report. Clin Orthop Relat Res 1982;206–8.

13. Spinner RJ, Sexton DJ, Goldner RD, Levin LS. Periprosthetic infections due to Mycobacterium tuberculosis in patients with no prior history of tuberculosis. J Arthroplasty 1996;11:217–22. doi: 10.1016/s0883-5403(05)80023-3

14. Cansü E, Erdogan F, Ulusam AO. Incision infection with Mycobacterium tuberculosis after total hip arthroplasty without any primary tuberculosis focus. J Arthroplasty 2011;26:505.e1-3. doi: 10.1016/j.arth.2009.11.025

15. Carbon C, Brion NV, Darcy M, Thomas M, Lamotte-Barrillon S. [Tuberculous infection of total hip prosthesis: report on two cases (author's transl)]. Ann Med Interne (Paris) 1981;132:124–5.

16. Carlsson AS, Sanzén L, Mikulowski P. Bilateral tuberculous infection of replaced hips--reactivation 54 years after infection in one knee. Acta Orthop Scand 1997;68:74–6. doi: 10.3109/17453679709003982

17. Carrega G, Bartolacci V, Burastero G, Finocchio GC, Ronca A, Riccio G. Prosthetic joint infections due to Mycobacterium tuberculosis: A report of 5 cases. Int J Surg Case Rep 2013;4:178–81. doi: 10.1016/j.ijscr.2012.11.011

18. Chazerain P, Desplaces N, Mamoudy P, Leonard P, Ziza JM. Prosthetic total knee infection with a bacillus Calmette Guerin (BCG) strain after BCG therapy for bladder cancer. J Rheumatol 1993;20:2171–2.

19. de Haan J, Vreeling AWJ, van Hellemondt GG. Reactivation of ancient joint tuberculosis of the knee following total knee arthroplasty after 61 years: a case report. Knee 2008;15:336–8. doi: 10.1016/j.knee.2008.03.004

20. De Nardo P, Corpolongo A, Conte A, Gentilotti E, Narciso P. Total hip replacement infected with Mycobacterium tuberculosis complicated by Addison disease and psoas muscle abscess: a case report. J Med Case Rep 2012 10;6:3. doi: 10.1186/1752-1947-6-3

21. Delrieu F, Slaoui O, Evrard J, Amor B, Postel M, Kerboull M. [Mycobacterial infection of the hip following total prosthesis. Study of 6 cases]. Rev Rhum Mal Osteoartic 1986;53:113–8.

22. Egües Dubuc C, Uriarte Ecenarro M, Errazquin Aguirre N, Belzunegui Otano J. Prosthesis infection by Mycobacterium tuberculosis in a patient with rheumatoid arthritis: A case report and literature review. Reumatol Clin 2014;10:347–9. doi: 10.1016/j.reuma.2014.02.003

23. Elzein FE, Haris M, Alolayan SS, Al Sherbini N. Total knee prosthesis infected with Mycobacterium tuberculosis. BMJ Case Rep 2017 7;2017:bcr2017220596, bcr-2017–220596. doi: 10.1136/bcr-2017-220596

24. Eskola A, Santavirta S, Konttinen YT, Tallroth K, Lindholm ST. Arthroplasty for old tuberculosis of the knee. J Bone Joint Surg Br 1988;70:767–9. doi: 10.1302/0301-620X.70B5.3192576

25. Fernández-Valencia JA, García S, Riba J. Presumptive infection of a total hip prosthesis by Mycobacterium tuberculosis: a case report. Acta Orthop Belg 2003;69:193–6.

26. Gale DW, Harding ML. Total knee arthroplasty in the presence of active tuberculosis. J Bone Joint Surg Br 1991;73:1006–7. doi: 10.1302/0301-620X.73B6.1955424

27. Goedertier W, Sioen W. Prosthetic joint infection due to Mycobacterium bovis 5-years after BCG-instillations. Acta Orthop Belg 2020;86:239–42.

28. Gomez E, Chiang T, Louie T, Ponnapalli M, Eng R, Huang DB. Prosthetic Joint Infection due to Mycobacterium bovis after Intravesical Instillation of Bacillus Calmette-Guerin (BCG). Int J Microbiol 2009;2009:527208. doi: 10.1155/2009/527208

29. Guerra CE, Betts RF, O'Keefe RJ, Shilling JW. Mycobacterium bovis osteomyelitis involving a hip arthroplasty after intravesicular bacille Calmette-Guérin for bladder cancer. Clin Infect Dis 1998;27:639–40. doi: 10.1086/514714

30. Harwin SF, Banerjee S, Issa K, Kapadia BH, Pivec R, Khanuja HS, et al. Tubercular prosthetic knee joint infection. Orthopedics 2013;36:e1464-1469. doi: 10.3928/01477447-20131021-35

31. Hattrup SJ, Bhagia UT. Shoulder arthroplasty complicated by mycobacterium tuberculosis infection: a case report. J Shoulder Elbow Surg 2008;17:e5-7. doi: 10.1016/j.jse.2008.01.146

32. Hecht RH, Meyers MH, Thornhill-Joynes M, Montgomerie JZ. Reactivation of tuberculous infection following total joint replacement. A case report. J Bone Joint Surg Am 1983;65:1015–6.

33. Hugate R, Pellegrini VD. Reactivation of ancient tuberculous arthritis of the hip following total hip arthroplasty: a case report. J Bone Joint Surg Am 2002;84:101–5. doi: 10.2106/00004623-200201000-00015

34. Jitmuang A, Yuenyongviwat V, Charoencholvanich K, Chayakulkeeree M. Rapidly-growing mycobacterial infection: a recognized cause of early-onset prosthetic joint infection. BMC Infect Dis 2017 28;17:802. doi: 10.1186/s12879-017-2926-3

35. Johnson R, Barnes KL, Owen R. Reactivation of tuberculosis after total hip replacement. J Bone Joint Surg Br 1979;61-B:148–50. doi: 10.1302/0301-620X.61B2.438263

36. Kadakia AP, Williams R, Langkamer VG. Tuberculous infection in a total knee replacement performed for medial tibial plateau fracture: a case report. Acta Orthop Belg 2007;73:661–4.

37. Kaya M, Nagoya S, Yamashita T, Niiro N, Fujita M. Peri-prosthetic tuberculous infection of the hip in a patient with no previous history of tuberculosis. J Bone Joint Surg Br 2006;88:394–5. doi: 10.1302/0301-620X.88B3.17006

38. Khater FJ, Samnani IQ, Mehta JB, Moorman JP, Myers JW. Prosthetic joint infection by Mycobacterium tuberculosis: an unusual case report with literature review. South Med J 2007;100:66–9. doi: 10.1097/01.smj.0000232972.50186.4c

39. Klein GR, Jacquette GM. Prosthetic knee infection in the young immigrant patient--do not forget tuberculosis! J Arthroplasty 2012;27:1414.e1-4. doi: 10.1016/j.arth.2011.09.020

40. Krappel FA, Harland U. Failure of osteosynthesis and prosthetic joint infection due to Mycobacterium tuberculosis following a subtrochanteric fracture: a case report and review of the literature. Arch Orthop Trauma Surg 2000;120:470–2. doi: 10.1007/s004029900091

41. Kreder HJ, Davey JR. Total hip arthroplasty complicated by tuberculous infection. J Arthroplasty 1996;11:111–4. doi: 10.1016/s0883-5403(96)80169-0

42. Langlois ME, Ader F, Dumistrescu O, Servien E, Saison J, Ferry T, et al. Mycobacterium bovis prosthetic joint infection. Med Mal Infect 2016;46:445–8. doi: 10.1016/j.medmal.2016.07.005

43. Lara-Oya A, Liébana-Martos MC, Rodríguez-Granger J, Sampedro-Martínez A, Aliaga-Martínez L, Gutierrez-Fernández J, et al. [Tuberculous prosthetic knee joint infection: a case report and literature review]. Rev Esp Quimioter 2016;29:214–9.

44. Leach WJ, Halpin DS. Mycobacterium bovis infection of a total hip arthroplasty: a case report. J Bone Joint Surg Br 1993;75:661–2. doi: 10.1302/0301-620X.75B4.8331128

45. Lederman E, Kweon C, Chhabra A. Late Mycobacterium tuberculosis infection in the shoulder of an immunocompromised host after hemiarthroplasty: a case report. J Bone Joint Surg Am 2011 15;93:e67(1-4). doi: 10.2106/JBJS.J.00710

46. Lee C-L, Wei Y-S, Ho Y-J, Lee C-H. Postoperative Mycobacterium tuberculosis infection after total knee arthroplasty. Knee 2009;16:87–9. doi: 10.1016/j.knee.2008.09.006

47. Levin ML. Miliary tuberculosis masquerading as late infection in total hip replacement. Md Med J 1985;34:153–5.

48. Lin E, Oliver S, Caspi I, Ezra E, Bubis JJ, Nerubay J. Hip arthroplasty in quiescent mycobacterial infection of hip. Orthop Rev 1986;15:232–6.

49. Lo CKL, Chen L, Varma S, Wood GCA, Grant J, Wilson EW. Management of Mycobacterium tuberculosis Prosthetic Joint Infection: 2 Cases and Literature Review. Open Forum Infect Dis 2021;8:ofab451. doi: 10.1093/ofid/ofab451

50. Lusk RH, Wienke EC, Milligan TW, Albus TE. Tuberculous and foreign-body granulomatous reactions involving a total knee prosthesis. Arthritis Rheum 1995;38:1325–7. doi: 10.1002/art.1780380921

51. Mahale YJ, Aga N. Implant-associated mycobacterium tuberculosis infection following surgical management of fractures: a retrospective observational study. Bone Joint J 2015;97-B:1279–83. doi: 10.1302/0301-620X.97B9.35227

52. Maricevic A, Dogas Z, Goic-Barisić I, Barisić I. Reactivation of tuberculosis after total hip replacement - 58 years after primary infection. Wien Klin Wochenschr 2008;120:642–3. doi: 10.1007/s00508-008-1006-5

53. Marmor M, Parnes N, Dekel S. Tuberculosis infection complicating total knee arthroplasty: report of 3 cases and review of the literature. J Arthroplasty 2004;19:397–400. doi: 10.1016/j.arth.2003.10.015

54. Marschall J, Evison J-M, Droz S, Studer UC, Zimmerli S. Disseminated tuberculosis following total knee arthroplasty in an HIV patient. Infection 2008;36:274–8. doi: 10.1007/s15010-007-7011-1

55. McCullough CJ. Tuberculosis as a late complication of total hip replacement. Acta Orthop Scand 1977;48:508–10. doi: 10.3109/17453677708989739

56. McLaughlin RE, Allen JR. Total hip replacement in the previously infected hip. South Med J 1977;70:573–5. doi: 10.1097/00007611-197705000-00022

57. Metayer B, Menu P, Khatchatourian L, Preuss P, Dauty M, Fouasson-Chailloux A. Prosthetic joint infection with pseudo-tumoral aspect due to Mycobacterium bovis infection after Bacillus-Calmette-Guerin therapy. Ann Phys Rehabil Med 2018;61:62–4. doi: 10.1016/j.rehab.2017.08.001

58. Mete B, Yemisen N, Aydin S, Babacan M, Ozaras R, Erdogan F, et al. An unusual cause of prosthetic joint infection:: Mycobacterium tuberculosis. Journal of Microbiology and Infectious Diseases 2012;2:72–5. doi: 10.5799/AHINJS.02.2012.02.0046

59. Meyssonnier V, Zeller V, Malbos S, Heym B, Lhotellier L, Desplaces N, et al. Prosthetic joint infections due to Mycobacterium tuberculosis: A retrospective study. Joint Bone Spine 2019;86:239–43. doi: 10.1016/j.jbspin.2018.09.008

60. Mouterde P, Deburge A. [Tuberculous infection after total hip replacement. Report of a case (author's transl)]. Rev Chir Orthop Reparatrice Appar Mot 1978;64:171–4.

61. Moya Megías R, Fernández Roldán C, Constán Rodríguez J, Javier Martínez MR. Tuberculosis on joint prosthesis as a form of presentation of miliary tuberculosis. Enferm Infecc Microbiol Clin (Engl Ed) 2023;41:441–2. doi: 10.1016/j.eimce.2023.04.013

62. Neogi DS, Kumar A, Yadav CS, Singh S. Delayed periprosthetic tuberculosis after total knee replacement: is conservative treatment possible? Acta Orthop Belg 2009;75:136–40.

63. Nguyen M-VH, Giordani MM, Thompson GR. The double-edged sword - prosthetic joint infection following BCG treatment for bladder cancer: a case report. BMC Infect Dis 2019 18;19:331. doi: 10.1186/s12879-019-3951-1

64. Olsson SS. [Tuberculous infection after hip replacement]. Lakartidningen 1981 29;78:1890–1.

65. Patel A, Elzweig J. Mycobacterium bovis prosthetic joint infection following intravesical instillation of BCG for bladder cancer. BMJ Case Rep 2019 18;12:e231830. doi: 10.1136/bcr-2019-231830

66. Perez-Jorge C, Valdazo-Rojo M, Blanco-Garcia A, Esteban-Moreno J. Mycobacterium tuberculosis as cause of therapeutic failure in prosthetic joint infections. Enferm Infecc Microbiol Clin 2014;32:204–5. doi: 10.1016/j.eimc.2013.04.022

67. Reigstad O, Siewers P. A total hip replacement infected with mycobacterium bovis after intravesicular treatment with Bacille-Calmette-Guérin for bladder cancer. J Bone Joint Surg Br 2008;90:225–7. doi: 10.1302/0301-620X.90B2.20038

68. Rispler DT, Stirton JW, Gilde AK, Kane KR. Mycobacterium bovid infection of total knee arthroplasty after bacille Calmette-Guérin therapy for bladder cancer. Am J Orthop (Belle Mead NJ) 2015;44:E46-48.

69. Riste M, Davda P, Smith EG, Wyllie DH, Dedicoat M, Jog S, et al. Prosthetic hip joint infection by Bacillus Calmette-Guerin therapy following intravesical instillation for bladder cancer identified using whole-genome sequencing: a case report. BMC Infect Dis 2021 5;21:151. doi: 10.1186/s12879-021-05831-3

70. Segal A, Krauss ES. Infected total hip arthroplasty after intravesical bacillus Calmette-Guérin therapy. J Arthroplasty 2007;22:759–62. doi: 10.1016/j.arth.2006.07.010

71. Seng P, Honnorat E, Loffeier V, Drancourt M, Stein A. Mycobacterium tuberculosis and prosthetic joint infection. Lancet Infect Dis 2016;16:894. doi: 10.1016/S1473-3099(16)30149-9

72. Shanbhag V, Kotwal R, Gaitonde A, Singhal K. Total hip replacement infected with Mycobacterium tuberculosis. A case report with review of literature. Acta Orthop Belg 2007;73:268–74.

73. Srivastava A, Ostrander J, Martin S, Walter N. Mycobacterium bovis infection of total hip arthroplasty after intravesicular bacille Calmette-Guérin therapy. Am J Orthop (Belle Mead NJ) 2011;40:E226-228.

74. Stern R, Roscoe C, Misch EA. Mycobacterium bovis BCG osteoarticular infection complicating immune therapy for bladder cancer: a case report. J Bone Jt Infect 2021;6:107–10. doi: 10.5194/jbji-6-107-2021

75. Storandt M, Nagpal A. Prosthetic joint infection: an extremely rare complication of intravesicular BCG therapy. BMJ Case Rep 2019 10;12:e232809. doi: 10.1136/bcr-2019-232809

76. Su JY, Huang TL, Lin SY. Total knee arthroplasty in tuberculous arthritis. Clin Orthop Relat Res 1996;181–7. doi: 10.1097/00003086-199602000-00024

77. Tekin Koruk S, Sipahioğlu S, Calişir C. Periprosthetic tuberculosis of the knee joint treated with antituberculosis drugs: a case report. Acta Orthop Traumatol Turc 2013;47:440–3. doi: 10.3944/aott.2013.2511

78. Tokumoto JI, Follansbee SE, Jacobs RA. Prosthetic joint infection due to Mycobacterium tuberculosis: report of three cases. Clin Infect Dis 1995;21:134–6. doi: 10.1093/clinids/21.1.134

79. Ueng WN, Shih CH, Hseuh S. Pulmonary tuberculosis as a source of infection after total hip arthroplasty. A report of two cases. Int Orthop 1995;19:55–9. doi: 10.1007/BF00184916

80. Uhel F, Corvaisier G, Poinsignon Y, Chirouze C, Beraud G, Grossi O, et al. Mycobacterium tuberculosis prosthetic joint infections: A case series and literature review. J Infect 2019;78:27–34. doi: 10.1016/j.jinf.2018.08.008

81. Uppal S, Garg R. Tubercular infection presenting as sinus over ankle joint after knee replacement surgery. J Glob Infect Dis 2010;2:71–2. doi: 10.4103/0974-777X.59257

82. Upton A, Woodhouse A, Vaughan R, Newton S, Ellis-Pegler R. Evolution of central nervous system multidrug-resistant Mycobacterium tuberculosis and late relapse of cryptic prosthetic hip joint tuberculosis: complications during treatment of disseminated isoniazid-resistant tuberculosis in an immunocompromised host. J Clin Microbiol 2009;47:507–10. doi: 10.1128/JCM.01473-08

83. Van Le T, Duong TB, Hien KQ, Ton QNQ, Huyn T, Binh TP, et al. Two-stage revision for treatment of tuberculous prosthetic hip infection: an outcome analysis. Eur J Orthop Surg Traumatol 2023;33:645–51. doi: 10.1007/s00590-022-03317-9

84. Veloci S, Mencarini J, Lagi F, Beltrami G, Campanacci DA, Bartoloni A, et al. Tubercular prosthetic joint infection: two case reports and literature review. Infection 2018;46:55–68. doi: 10.1007/s15010-017-1085-1

85. von Keudell A, Nathavitharana R, Yassa D, Abdeen A. An unusual pathogen for prosthetic joint infection. Lancet Infect Dis 2016;16:506. doi: 10.1016/S1473-3099(15)00398-9

86. Walczak H. [ON THE TREATMENT OF DIFFERENT TYPES OF RHEUMATIC DISEASES IN PULMONARY TUBERCULOSIS AND LUNG TUMORS]. Z Tuberk Erkr Thoraxorg 1964;121:79–82.

87. Wang P-H, Shih K-S, Tsai C-C, Wang H-C. Pulmonary tuberculosis with delayed tuberculosis infection of total knee arthroplasty. J Formos Med Assoc 2007;106:82–5. doi: 10.1016/S0929-6646(09)60221-7

88. Williams A, Arnold B, Gwynne-Jones DP. Mycobacterium bovis infection of total hip arthroplasty after intravesicular Bacillus Calmette-Guérin. Arthroplast Today 2019;5:416–20. doi: 10.1016/j.artd.2019.08.004

89. Wolfgang GL. Tuberculosis joint infection following total knee arthroplasty. Clin Orthop Relat Res 1985;162–6.

90. Wray CC, Roy S. Arthroplasty in tuberculosis of the knee. Two cases of missed diagnosis. Acta Orthop Scand 1987;58:296–8. doi: 10.3109/17453678709146492

91. Zeiger LS, Watters W, Sherk H. Scintigraphic detection of prosthetic joint and soft tissue sepsis secondary to tuberculosis. Clin Nucl Med 1984;9:638–9. doi: 10.1097/00003072-198411000-00008
